# Chitinolytic functions in actinobacteria: ecology, enzymes, and evolution

**DOI:** 10.1007/s00253-018-9149-4

**Published:** 2018-06-21

**Authors:** Marie-Ève Lacombe-Harvey, Ryszard Brzezinski, Carole Beaulieu

**Affiliations:** Département de biologie, Université de Sherbrooke, Sherbrooke, QC J1K 2R1 Canada

**Keywords:** Actinomycete, Chitin, Chitinase, Chitosan, Chitosanase, Glycosyl hydrolase, *Streptomyces*

## Abstract

Actinobacteria, a large group of Gram-positive bacteria, secrete a wide range of extracellular enzymes involved in the degradation of organic compounds and biopolymers including the ubiquitous aminopolysaccharides chitin and chitosan. While chitinolytic enzymes are distributed in all kingdoms of life, actinobacteria are recognized as particularly good decomposers of chitinous material and several members of this taxon carry impressive sets of genes dedicated to chitin and chitosan degradation. Degradation of these polymers in actinobacteria is dependent on endo- and exo-acting hydrolases as well as lytic polysaccharide monooxygenases. Actinobacterial chitinases and chitosanases belong to nine major families of glycosyl hydrolases that share no sequence similarity. In this paper, the distribution of chitinolytic actinobacteria within different ecosystems is examined and their chitinolytic machinery is described and compared to those of other chitinolytic organisms.

**Electronic supplementary material:**

The online version of this article (10.1007/s00253-018-9149-4) contains supplementary material, which is available to authorized users.

## Introduction

Actinobacteria are Gram-positive bacteria possessing relatively large genomes, often over 5 megabases, characterized by high G+C contents (Lewin et al. 2016). Several of them, including the abundant members of the *Streptomyces* genus, exhibit a complex life cycle producing substrate mycelium, aerial mycelium and spores. They are widely distributed in both terrestrial and aquatic ecosystems. Although members of this large phylum exist as free-living saprophytes, several of them can live inside tissues or organs as commensal or symbiotic partners of plants (Matsumoto and Takahashi 2017; Santi et al. 2013), insects (Kaltenpoth 2009; Matarrita-Carranza et al. 2017; Seipke et al. 2012), aquatic animals (Dharmaraj 2010; Ian et al. 2014; Mahmoud and Kalendar 2016) as well as terrestrial animals and human beings (Hugon et al. 2015). Although actinobacteria can infect plants (Hogenhout and Loria 2008) or cause animal and human diseases (Luo et al. 2014; McNeil and Brown 1994; Vázquez-Boland et al. 2013), the proportion of pathogenic species in the group of bacteria is low.

Actinobacteria play an essential role in carbon cycling especially in regard to the solubilization of plant and fungal cell walls, as well as insect cuticles and crustacean shells (Chater 2016). They secrete a wide range of extracellular proteins that represent a source of enzymes of industrial interest (Mukhtar et al. 2017). *Streptomyces coelicolor* genome encodes indeed over 800 putative secreted proteins (van der Meij et al. 2017) and the plant pathogen *Str. scabies*, when grown in the presence of potato periderm, produces over 200 different extracellular proteins which mostly are glycosyl hydrolases (Beaulieu et al. 2016). In nature, actinobacterial extracellular enzymes are involved, among others, in the degradation of complex or recalcitrant biopolymers such as lignocellulose (Book et al. 2014; Goodfellow 1983; Padilla-Reynaud et al. 2015; Wang et al. 2016), keratin (Mukhtar et al. 2017), suberin (Beaulieu et al. 2016) and chitin (Beier and Bertilsson 2013). This review is focused on the degradation of chitin and chitosan with emphasis on natural environments, molecular families of the genes, and proteins involved in these processes and their evolutionary relationships.

## Chitinolytic properties of actinobacteria

Chitin, a β-1,4-linked polysaccharide of *N*-acetylglucosamine, is the second most abundant biopolymer in nature, being associated with fungal cell walls (Rane and Hoover 1992), crustacean exoskeletons, insect cuticles and nematode egg shells (Shinya and Fukamizo 2017). Chemical or enzymatic *N*-deacetylation of chitin gives rise to chitosan. There is, however, no clear delimitation between chitin and chitosan in regard to their degrees of acetylation. The distribution of *N*-acetyl glucosamine and glucosamine residues within the polymer chains determines the types of enzymes required for the hydrolysis of these substrates (Rinaudo 2006).

Chitinolytic^1^ enzymes are produced by a large spectrum of bacteria and eukaryotes including plants and animals (Adrangi and Faramarzi 2013) but in prokaryotes, actinobacteria are among the best chitin decomposers. They can utilize chitin or chitosan as carbon and nitrogen sources (Beier and Bertilsson 2013) and have impressive sets of enzymes for the degradation of chitin. Chitin, which is omnipresent in nature, represents a marker of the environmental nutrient status for streptomycetes. The environmental signal, *N*-acetyl glucosamine, the monomeric form of chitin, is metabolized inside the cell to glucosamine-6P. In *Str. coelicolor*, this pathway influences directly the global regulator DasR that controls chitinolysis, development, antibiotic biosynthesis and siderophore production (Craig et al. 2012; van der Meij et al. 2017).

Since the early 1960s, chitin-containing media have been routinely used for the selective isolation of actinobacteria (Lingappa and Lockwood 1961). Plating of different soil (Lingappa and Lockwood 1961) or freshwater dilutions (Hsu and Lockwood 1975), onto a medium containing colloid chitin as carbon and nitrogen sources, allowed the recovery of actinobacterial colonies over those of other bacterial or fungal colonies. When grown on chitin-containing solid medium, clearing zone surrounding actinobacterial colonies reveals that their growth depends, at least partially, on their ability to solubilize chitin. Nevertheless, there are organisms possessing genes coding for chitinolytic enzymes but not growing on chitin-containing culture media. Notable examples include the erythromycin producer *Saccharopolyspora erythraea* and the potent cellulose degrader *Thermobifida fusca* (Gaber et al. 2016; Liao et al. 2014).

Chitin agar is still used up to the present day for selective isolation of free living actinobacteria as well as actinobacteria interacting with plants (Golinska et al. 2015) or animals (Arango et al. 2016; Zhang et al. 2006). It has been also used to recover chitinolytic bacteria from lake sediments of Antarctica (Xiao et al. 2005). The microflora of a sediment core that spanned approximately 1600 years has been affected by penguin guano which contains high amounts of chitin from krill and squid, the main penguin food sources. Xiao et al. (2005) isolated only six bacterial strains from this sediment core but three of them belonged to the *Actinobacteria* taxon. Sequences of the chitinase genes in these strains shared about 80% identity with *chiC* from *Str. coelicolor* (Xiao et al. 2005). While chitin agar allows the isolation of common actinobacterial genera, pretreatments of the environmental samples or enrichment procedures prior to selection on chitin agar have, however, been proposed to promote isolation of rare actinobacteria (Subramani and Aalbersberg 2013; Wohl and McArthur 1998). Chitin has also been used as selective substrate in a resuscitation procedure of bacteria trapped in Antarctic permafrost (Manucharova et al. 2016). When dormant microbial communities from Antarctic permafrost rocks (7500-year-old sediments) were reactivated by rehydrating the cells and adding methylresorcinol and yeast extract, the biomass of the metabolically active prokaryotic population in permafrost sediment reached 10% of the total biomass. However, selection on chitin after this reactivation step resulted in an increased portion of metabolically active biomass, from 10 to 50%. Actinobacteria was the dominant bacterial group of this permafrost rocks community (69% of total cells); biomass of the metabolically active actinobacteria showing a 25-fold increase after chitinolysis initiation (Manucharova et al. 2016).

Abundance of chitinolytic actinobacteria in a lake and soils of the same lake basin in Central Poland (Swiontek Brzezinska et al. 2009) has been compared using chitin agar as selective growth medium. This study demonstrated that chitinolytic actinobacteria were not only more abundant in soil than in water, but actinobacteria from soils also exhibited higher chitinolytic activity than lake isolates. The authors attributed the differences in both number and activity to higher accumulation of chitinous material in terrestrial ecosystems.

Considering the wide distribution of chitinolytic genes among prokaryotes (Nguyen et al. 2018), the relative selectivity of chitin agar for actinobacteria is, however, seen by some authors as a “plate count anomaly” (Kielak et al. 2013). Indeed, the presence of chitin in the environment did not necessarily coincide with a predominance of actinobacteria within the microbial community, especially when the structure of microbial community was analyzed using biomolecular tools. The effect of chitin amendment on the taxonomical and functional structure of bacterial communities has been studied by several groups under natural or microcosm conditions. For example, a recent metatranscriptomics study, where different biopolymers were added to peat from an acidic peatland in North Russia, revealed no global effect of chitin enrichment on the actinobacterial population, although chitin stimulated development of streptomycetes, which were only rarely detected in the unamended peat (Ivanova et al. 2016). Amendment of chitin in lake water induces an increase in abundance of the acI tribe of actinobacteria (Beier and Bertilsson 2011). Lineage acI was originally defined in 2004 by Warnecke et al. (2004). In surface lake waters, actinobacteria often constitute the dominant phylum, representing up to 70% of the bacterioplankton and where the planktonic acI actinobacterial tribe is the most represented (Warnecke et al. 2004; Beier and Bertilsson 2011; Garcia et al. 2013). A putative chitinase-encoding gene has been detected in the genome of an acI lineage representative (Garcia et al. 2013), what remains somewhat in contrast with the analysis of the first four entirely sequenced genomes of acI tribe members in which genes related to chitin degradation and metabolism were not found (Kang et al. 2017). The contradiction is only apparent as the acI tribe could include as much as hundreds, perhaps thousands of different members and diversity of specialized carbon utilization functions in their small genomes was already observed (Kang et al. 2017). While the acI tribe positively responded to chitin amendment (Beier and Bertilsson 2011), these bacteria did not appear to colonize chitin particles and were exclusively detected as free-living cells in the water lake amended with chitin material. The ecological interest for bacteria with a strictly planktonic lifestyle to produce extracellular enzymes spatially distant from the site of the enzymatic activity could be questioned (Beier and Bertilsson 2011). These authors thus suggested that the increase in the population of the acI tribe did not result from the expression of a chitin hydrolysis system but rather from the uptake of small chitin hydrolysis products released by the action of bacteria colonizing chitinous particles. According to Eckert et al. (2013), the physiological adaptation of the acI tribe to the sequestration of *N*-acetyl glucosamine is especially beneficial during vernal phytoplankton blooms. As an important class of grazing-protected bacteria in lakes, the acI tribe would benefit from a higher availability of organic compounds arising from the lysis of prey bacteria by bacterivorous protozoa, and especially of *N*-acetyl glucosamine that is not only a monomer of chitin but also a main constituent of the bacterial cell wall (Eckert et al. 2013).

Divergent effects of chitin amendment on soil actinobacterial communities have been reported in literature. This contradictory effects may in fact depend on several environmental factors such as temperature (Manucharova et al. 2011), pH (Kielak et al. 2013), type of soil (Swiontek Brzezinska et al. 2010a), chitin source (Swiontek Brzezinska et al. 2010b), humidity level (Vorob’ev et al. 2007), etc. Cretoiu et al. (2014), who compared the bacterial diversity of an agricultural soil supplemented or not with chitin, showed that 9 months after the amendment, the abundance of actinobacteria was significantly reduced in the chitin-amended field soil. The decrease was especially important for the Streptomycetaceae and Streptosporangiaceae, even if both groups are known as good chitinase producers. Kielak et al. (2013), who examined over a 60-day-period the bacterial populations of agricultural soil amended with ground shrimp shells showed that both the actinobacterial 16S rRNA gene quantification and the actinobacterium-related *chiA* gene decreased in the presence of chitin. However, when an agricultural soil was amended twice with chitinous material, a first time in spring 2007 and a second time in fall 2009, a significant increase in actinobacteria abundance in the chitin-amended soil was observed 8 months after the second treatment and then, persisted over time (Cretoiu et al. 2013). It thus appears that soil actinobacteria might exhibit slower responses to chitin stimulus than other bacterial groups (Kielak et al. 2013). The response of the soil actinobacterial community to chitin enrichment also appears to depend on chitin concentrations. Jacquiod et al. (2013), who compared the effect of two doses of chitin (2 and 20 mg/g soil) in a soil microcosm experiment, established that the lowest chitin concentration did not significantly modify the soil bacterial community structure while an effect was observed at the highest concentration. Under high chitin concentration, chitin leads to an increase of the actinobacterial population. However, this increase only depends on a fraction the actinobacterial population since the hybridization signals on phylochips corresponding to this phylum decreased; whereas some actinobacterial genera (*Aeromicrobium*, *Microbacterium*, *Nocardioides*, and *Solirubrobacter*) were detected only in the presence of chitin (Jacquiod et al. 2013).

Soil or compost amendment with chitinous material can be viewed as an indirect biocontrol practice in agriculture since it often increases suppressiveness of soil towards plant pathogens. This effect correlates with a raise of the actinobacterial population (Bell et al. 1998; Cretoiu et al. 2013; Debode et al. 2016; Labrie et al. 2001). Several chitinolytic actinobacterial strains have indeed been found to protect plants against plant diseases or to promote their growth. Some of these chitinolytic strains can even adopt an endophytic lifestyle after their entry in plant tissues through lateral root emergence areas, other natural openings or wounds (Santi et al. 2013). Chitinolytic actinobacterial strains are currently used as active ingredients of commercial fungicides (Doumbou et al. 2001; Rey and Dumas 2017). Although antibiosis has been shown to contribute to plant protection (Doumbou et al. 2001), chitinases produced by the actinobacteria are also thought to participate in antagonistic interactions with pathogenic fungi (El-Tarabily et al. 2000; Rey and Dumas 2017; Veliz et al. 2017). Chitinases could be especially efficient in lysis of fungal hyphal tips since the cell wall in this region is composed of chitin only in contrast to distal region of fungal hyphae where chitin is intermixed with β-glucans and proteins (Gooday 1995; Theis and Stahl 2004). Chitinolytic actinobacteria also contribute to plant health by promoting the association of plant roots with arbuscular mycorrhizal fungi (AMF). Indeed, chitin decomposing actinobacteria closely adhere to AMF spore walls (Selvakumar et al. 2016) and the release of short chitin oligomers by chitinases leads to the activation of a signalling pathway involved in the first steps of AMF root colonization (Genre et al. 2013). As an interesting way to introduce a chitinolytic actinobacterium in a plant production system, Jobin et al. (2005) proposed the encapsulation of the microorganisms into chitosan beads. After their introduction in the environment, the beads could serve as carbon and nitrogen sources for the chitinolytic actinobacterium thus helping its implementation in the plant environment while the chitosan bead degradation would lead to the liberation of chitooligosaccharides that could act as elicitors of plant immunity responses (Jobin et al. 2005).

## Overview of actinobacterial enzymes involved in chitinolysis

In contrast with cellulose degradation, a modest number of enzyme families are involved in chitinolysis (Talamantes et al. 2016). As for other polymers, a combination of endo- and exo-acting hydrolases assisted by monooxygenases is required. Actinobacterial endo-chitinases are grouped in GH18 and GH19 families which are essentially monospecific, while chitosanases are found in monospecific families GH46 and GH75 or polyspecific families GH5 and GH8. Enzymes with chitinase or chitosanase activities have been also identified in families GH23, GH48 and GH3, GH7, GH80, respectively (Coutinho and Henrissat 1999), but their presence in actinobacteria has not been documented so far (Nguyen et al. 2018). The endo-hydrolases generate short oligomers (essentially dimers, occasionally trimers and longer oligomers) as shown by the pioneering work by Reynolds (1954) for a chitinolytic *Str. griseus* strain.

Chitin or chitosan hydrolysis has to be completed by exohydrolases. Actinobacterial exohydrolases—exo-β-*N*-acetylglucosaminidases (GlcNAcase; former name: chitobiase) belong to families GH3 and GH20 while exo-β-glucosaminidases (GlcNase) represent a subfamily within the GH2 family (Côté et al. 2006). Minor numbers of enzymes with GlcNAcase or GlcNase activities were identified in families GH5, GH84, GH116 or GH9, GH35, respectively, but their presence in actinobacteria is not documented so far in the CAZy database (Lombard et al. 2014).

Streptomycetes and related genera have, as a rule, many chitinases paralogs, especially those belonging to family GH18. The presence of ten or more GH18 genes in their genomes is not exceptional. In a large proportion of these genes, the presence of carbohydrate-binding modules (CBM) is also observed (Henrissat and Davies 2000; Lombard et al. 2014). The diversity of chitinases produced by a single actinobacterial strain has been first investigated in *Str. olivaceoviridis* (reviewed by Schrempf 2001, 2017) and *Str. lividans* (Miyashita et al. 1991). These pre-genomic era studies showed that individual chitinases are often present in multiple forms in chitin-based culture media, as the full-length proteins generate their truncated forms by proteolytic cleavage. In *Str. lividans*, a single cloned chitinase gene directed the production of two enzyme forms (Fujii and Miyashita 1993). For Chi01 chitinase of *Str. olivaceoviridis,* the larger 59 kDa enzyme, which included a CBD, hydrolyzed crystalline chitin more efficiently than the shorter 47 kDa form lacking the CBD (Blaak and Schrempf 1995). The generation of multiple enzyme forms from a single gene is a common strategy used by streptomycetes to increase the enzymatic diversity of their chitinolytic system.

The most extensive study of chitinase diversity in actinobacteria was dedicated to the model strain *Str. coelicolor* A3(2) whose genome encodes 11 GH18 and two GH19 members (Kawase et al. 2006; Saito et al. 1999). The large majority of these genes (11/13) includes CBDs. Kawase et al. (2006) compared the properties of three GH18 and one GH19 chitinases highly expressed in the presence of chitin. They differed in their response to pH and temperature variations. The GH19 chitinase (Chi19F) emerged as a potent antifungal agent and hydrolyzed efficiently soluble chitin oligomers while the GH18 enzymes (Chi18aC, Chi18aD, and Chi18bA) markedly preferred crystalline chitin forms, Chi18aC being significantly more efficient than the other two. It is then suggested that GH18 chitinases play a major role in the degradation of various chitin forms while the GH19 enzyme could play a minor role in degradation or even be dispensable. On the other hand, the GH19 enzyme could play an essential role in the interactions between *Streptomyces* and fungi (Kawase et al. 2006).

Endohydrolases dedicated to chitosan hydrolysis offer quite a different portrait. Only a small minority of genes include a CBD. The GH46 family is the most representative for *Streptomyces* and related genera (Viens et al. 2015b). For this family, as well as GH75, most genomes include only one or two genes. Globally, the number of chitosanase genes is much lower than that of chitinases, what is probably related to the fact that chitosan is much less abundant than chitin in nature. The diversity of chitosanases in a single strain was most extensively studied for *Kitasatospora setae*, whose genome encodes three GH46 chitosanases: Csn1, Csn2, and Csn3 (Zitouni et al. 2017). One of them (Csn2) includes a CBD and was produced both in full-length and truncated forms. In all aspects, Csn1 and truncated Csn2 were very similar, while full-length Csn2 was distinct by its higher relative activity on chitosan complexed with polyphosphate. On the other hand, Csn3 had a much higher specific activity (per mg of protein) but was proportionally less active at low temperatures. Enzymes also differed by their relative activities against highly *N*-deacetylated chitosan, which was preferably hydrolyzed by Csn1 and Csn2, while Csn3 had no preference compared with moderately *N*-deacetylated chitosan (Zitouni et al. 2017).

As stated above, endo-acting enzymes dedicated to chitin hydrolysis are present in much larger numbers in actinobacteria that those involved in chitosan hydrolysis. The same can be observed with exo-hydrolases: while GH3 and GH20 GlcNAcases dedicated to complete the hydrolysis of chitin oligomers into monomers are widely distributed (usually several genes per genome), GH2 GlcNases involved in chitosan hydrolysis are present only in a minority of species (Côté et al. 2006). The degradation of chitin or chitosan into monomers by secreted exo-hydrolases is however not mandatory for their efficient utilization as nutrients, as actinobacteria possess specialized transport systems for oligomeric forms of chitin and chitosan. Most of these systems belong to the category of ABC transporters (Saito et al. 2007; Viens et al. 2015a; Xiao et al. 2002). Following the capture of oligomers, the hydrolysis into monomers is performed intracellularly (Saito et al. 2013; Viens et al. 2015a). The capacity to use oligomers directly can be considered as an advantage over competing microorganisms that are only able to use monomers, such as *Saccharopolyspora erythraea* which lacks the ABC transporter for chitin dimer transport (Liao et al. 2014). Actinobacteria can also use monomeric GlcNAc and GlcN, but many questions remain regarding the (possibly multiple) pathways for their transport and intracellular metabolism (reviewed by Urem et al. 2016).

The presence of a CBD in genes encoding exo-acting enzymes is rather exceptional. A CBD is however observed in the GlcNase from *Amycolatopsis orientalis* (Côté et al. 2006). Surprisingly, this CBD does not bind neither to polymeric nor to oligomeric chitosan forms. Instead, binding to uronic acid sugars—a component of bacterial cell wall was observed (Montanier et al. 2009). In vivo, this resulted in anchoring the CsxA protein to the extracytoplasmic compartment, likely promoting the degradation of chitosan molecules entering in close contact with the bacterial cell (Montanier et al. 2009).

The spectrum of proteins involved in chitinolysis is completed by the so-called chitin-binding proteins. As an example, CHB1 is a small protein extracellularly secreted by *Str. olivaceoviridis* in chitin-based media together with the chitinases. CHB1 targeted α-chitin filaments in vitro (Siemieniewicz et al. 2007). In vivo, CHB1 acted as a facilitator of the remodeling of chitin filaments and initiator of contacts between *Streptomyces* cells and fungal hyphae, then promoting a more efficient hydrolysis of chitin in the fungal cell wall (Siemieniewicz and Schrempf 2007). With the discovery of the enzymatic activity of these proteins, now called lytic polysaccharide monooxygenases (LPMOs) (Vaaje-Kolstad et al. 2010; reviewed by Agostoni et al. 2017), it was shown that the effects observed both in vitro and in vivo were not only due to the binding of LPMOs to chitin chains and fungal cell walls but essentially to their capacity to cleave chitin chains using a novel, oxidative mechanism. Actinobacterial LPMOs belong to family AA10, grouping enzymes acting either on chitin or cellulose. These enzymes require divalent copper ions for activity (Forsberg et al. 2014). One of the six putative AA10 family LPMO of *Str. griseus*, SgLPMO10F (SGR_6855), was shown to bind to both α- and β-chitin (although with a different affinity) and increased markedly the solubilization of both forms of chitin in synergy tests with several chitinases (Nakagawa et al. 2015). The LPMOs are now considered as important, if not essential contributors to chitin degradation and their use for biotechnological biomass conversion is also envisioned (Hemsworth et al. 2015).

Terrestrial actinobacteria appear to be among the best adapted prokaryotes to use chitin. Bai et al. (2016), who examined the genomes of 110 chitinolytic prokaryotes, determined that terrestrial actinobacteria have the highest number of chitinase genes, the highest diversity of associated carbohydrate-binding modules and the highest number of lytic polysaccharide monooxygenases.

## Regulation of chitinolytic gene expression

The inducibility of chitinases production by the presence of chitin in actinobacteria was first observed by Reynolds (1954) in *Str. griseus* and later confirmed by many authors. When the first sequences of chitinase genes from *Str. plicatus* and *Str. lividans* were determined (Miyashita and Fujii 1993; Robbins et al. 1992), it was noticed that they possessed short direct repeat sequences in the promoter segment, considered as possible binding sites for a regulatory protein. Indeed, Delic et al. (1992) showed that two promoter segments from *Str. plicatus* chitinase genes directed a chitin-inducible, glucose-repressible transcription of a reporter gene in the heterologous host *Str. lividans*. An electrophoretic gel mobility shift assay (EMSA) detected the presence of a protein able to bind to the promoter segment in a crude extract from cells grown in rich medium without chitin. Single-base mutations within the repeated sequences resulted in deregulated chitinase production (Ni and Westpheling 1997). All this suggested that the direct repeats are the binding site of a repressor-type regulator.

Several genes and proteins were proposed as regulators of chitinase gene expression, based on the effects of disruption of these genes on production of chitinases in various conditions. For instance, deletion of the *reg1* gene in *Str. lividans* (ortholog of SCO2232 in *Str. coelicolor*) resulted in production of chitinases in the absence of chitin and relieved chitinases production from glucose repression (Nguyen et al. 1997). Similarly, the disruption of the *chiR* gene (SCO5376) forming a two-component regulatory system together with *chiS* in *Str. coelicolor* resulted in substantially reduced transcription level of chitinases gene *chiC* both in the presence and absence of chitin (Homerová et al. 2002). Fujii et al. (2005) purified a protein able to bind to the promoter segment of the chitinase A gene in *Str. lividans*. After the determination of the *N*-terminal sequence of this protein, they cloned the corresponding *cpb1* gene (an ortholog of SCO4441). Disruption of *cpb1* resulted in a partial relief of chitinase production from glucose repression in the presence of chitin and had no effect on chitinase induction by chitin. However, specific binding to the direct repeats localized in the promoter region of chitinase genes could not be demonstrated for any of these regulatory proteins.

Such specific binding has been demonstrated for the protein DasR encoded by the SCO5231 gene in *Str. coelicolor* (Colson et al. 2007), first discovered in *Str. griseus* (Seo et al. 2002). Binding of DasR to promoter segments of several chitinase genes (*chiD*, *chiH*, *chiI*) or genes encoding chitin-binding proteins (SCO6345 and SCO7225) was demonstrated by EMSA. Binding occurred with regions having one or more operator sequences, renamed as *dre* (DasR-responsive elements). Binding was also observed with *dre* sequences synthesized as oligonucleotides (Colson et al. 2007). Disruption of *dasR* gene in *Str. coelicolor* had dramatic effects not only on chitinase genes expression but also on secondary metabolism and differentiation. DasR was revealed to be a global regulator, staying at the top of a control mechanism involving some 40 transcriptional regulators and influencing the expression of around 1200 genes (Świątek-Połatyńska et al. 2015). The *dre* consensus sequence was determined as a 16-bp motif A(G/C)TGGTCTAGACCA(G/C)T. A genome-wide analysis of DasR binding in vivo by the ChIP-on chip approach revealed that DasR binds to the promoters of a large proportion of genes involved in chitinase production and in the metabolism of the chitin monomer GlcNAc (Świątek-Połatyńska et al. 2015). Surprisingly, a transcriptomic experiment revealed only minor differences in transcription levels of chitinase genes between the wild-type and the *dasR*-deleted strains, indicating that additional levels of regulation are necessary for efficient transcription response to the presence of chitin. The role of DasR as global regulator has been reviewed recently (Romero-Rodríguez et al. 2015; Urem et al. 2016).

On the chitosanase side, a palindromic box was observed in the promoter segment of the first chitosanase gene sequenced in an actinobacteria (*Streptomyces* sp. N174) and was suggested to play a regulatory role (Masson et al. 1993). This sequence has been shown to be a target for a DNA-binding protein identified in the crude extract from cells of another efficient chitosanase producer, *Kitasatospora* sp. N106 (Dubeau et al. 2005). Through the analysis of many putative chitosanase genes, the consensus AGGAAANTTTCCT could be deduced. EMSA competition tests with oligonucleotides harboring single mutations showed that positions 5 and 9 in this sequence are the most important binding determinants (Dubeau et al. 2005).

Palindromic sequences corresponding to above consensus were identified in the genomes of *Str. lividans* and *Str. coelicolor* upstream from the chitosanase gene *csnA* (SCO0677) but also the SCO2657 gene encoding a putative repressor belonging to the ROK family (Titgemeyer et al. 1994) tentatively named *csnR* (Dubeau et al. 2011). The CsnR protein has been shown to bind in vitro to the palindromic boxes of both *csnA* and *csnR* genes. Binding in vitro was affected by the presence of chitosan oligomers, the (GlcN)_2_ dimer showing the strongest competition effect.

The *csnR* gene is localized at the beginning of a gene cluster composed of six genes (*csnREFGHK*) encoding an ABC transporter, a glycoside hydrolase from family GH4 and a putative saccharide kinase (Dubeau et al. 2011). The CsnEFG transporter was shown to be involved in the binding and transport of oligosaccharides derived from chitosan (Viens et al. 2015a). Disruption of the *csnR* gene resulted in a transcriptional derepression of the chitosanase gene *csnA* and of the *csnE-K* genes. CsnR is then a negative regulator of the chitosanase gene and of its own operon. The palindrome bound by CsnR was named “the CsnR-box”.

The *csnREFGHK* cluster is conserved in many actinomycete genomes. Most of these clusters could be putatively self-regulated as they have CsnR operators. Also, the CsnR-box is present in many chitosanase genes belonging not only to GH46 family but also GH2, GH5 and GH75 (Dubeau et al. 2011). Their importance for induction by chitosan has been shown in *Str. avermitilis* where three putative chitosanase genes with CsnR- boxes exhibited enhanced transcription in the presence of chitosan, while two other genes without boxes were not transcribed under the same conditions (Dubeau et al. 2011).

In *Str. coelicolor*, the pathways of chitin and chitosan hydrolysis and metabolism seem to be regulated by two distinct mechanisms dependent on DasR and CsnR, respectively. This separate regulation mode could be present in other actinobacteria as well: the sequences of genes discussed in this section reveal that *dre* elements are absent from genes related to chitosan degradation while CsnR boxes are not found in genes related to chitin metabolism (data not shown). Mining the available actinomycete genomes, we have however found one strain escaping from this rule: *Streptosporangium roseum*. While most genomes include, as average, only three to four genes with CsnR-box, we have identified in the genome of *Streptosporangium roseum*, using the RSAT tool (Thomas-Chollier et al. 2011), as much as 15 genes with CsnR boxes, possibly controlled by the CsnR ortholog Sros_5819. This putative regulon includes notably several genes encoding chitinases and chitin-binding proteins (Supplementary Fig. S1). An ortholog of DasR could not be identified in the genome of *Streptosporangium roseum* (data not shown). Thus, in this organism, chitin and chitosan metabolism could be more deeply integrated than in the other organisms studied so far.

## Molecular evolution of chitinolytic enzymes in actinobacteria

Although GH18 and GH19 endo-chitinases both catalyze the degradation of chitin, they share no sequence similarity and display two distinct structural folds (TIM-barrel fold and lysozyme-like fold, respectively) indicating independent evolutionary origins (Adrangi and Faramarzi 2013). It is generally believed that GH18 is an ancient gene family, as GH18 chitinases are widely represented in the three major kingdoms of life: archaea, prokaryotes and eukaryotes (Funkhouser and Aronson Jr 2007). Based on diversity, domain structure, and phylogenetic relationships, Karlsson and Stenlid (2009) divided the GH18 chitinases in three main clusters, A, B, and C. Chitinases from actinobacteria belong to all the three clusters. An alignment of 379 primary sequences revealed that cluster A includes bacterial, fungal, plant, animal and viral members, cluster B contains chitinases from bacteria, fungi, and plants while cluster C contains bacterial and archaean representatives. These data suggest that the differentiation of cluster A and B preceded the appearance of the eukaryotic lineage. Interestingly, the GH18 chitinases from *Str. coelicolor* are spread over all the three clusters. This illustrates the conclusion from this study that GH18 protein sequences do not cluster according to taxonomy (Karlsson and Stenlid 2009). Chitinases from *Str. coelicolor* belonging to cluster A are members of two different subgroups; subgroup A-II, which includes mainly proteins from actinobacteria, and subgroup A-VI, which is composed nearly exclusively of proteins from Gram-negative bacteria. In *Str. coelicolor*, gene *chiI* belongs to the latter subgroup. DNA sequence analysis of *chiI* gene with FramePlot software (Ishikawa and Hotta 1999) reveals that it has a high G+C content (64.9%) with a typically actinobacterial codon distribution; i.e., no sign of horizontal gene transfer (HGT) from Gram-negative bacteria. The extensive analysis by Karlsson and Stenlid (2009) also highlighted the fact that GH18 bacterial and fungal chitinases genes do not form monophyletic groups, in opposition to earlier suggestions (Gan et al. 2007; Wang et al. 2004).

In contrast to the widespread distribution of GH18 family members, GH 19 chitinases are found only in plants, bacteria, and viruses. Based on identification and selective absence or presence of conserved sequences motifs across subgroups within the GH19 family, Udaya Prakash et al. (2010) proposed that actinobacterial GH19 genes were initially obtained from plants. Moreover, the study suggests that the rare GH19 genes found in arthropods were acquired from actinobacteria. This corroborated the early idea that *Streptomyces* GH19 chitinases were acquired from plants by HGT (Watanabe et al. 1999). Based on the distribution of GH19 chitinases in actinobacteria and phylogenetic relationships it was suggested that a GH19 chitinase gene was first acquired by an ancestor of the *Streptomycineae* and spread among actinobacteria by multiple HGT events (Kawase et al. 2004).

**Fig. 1:**
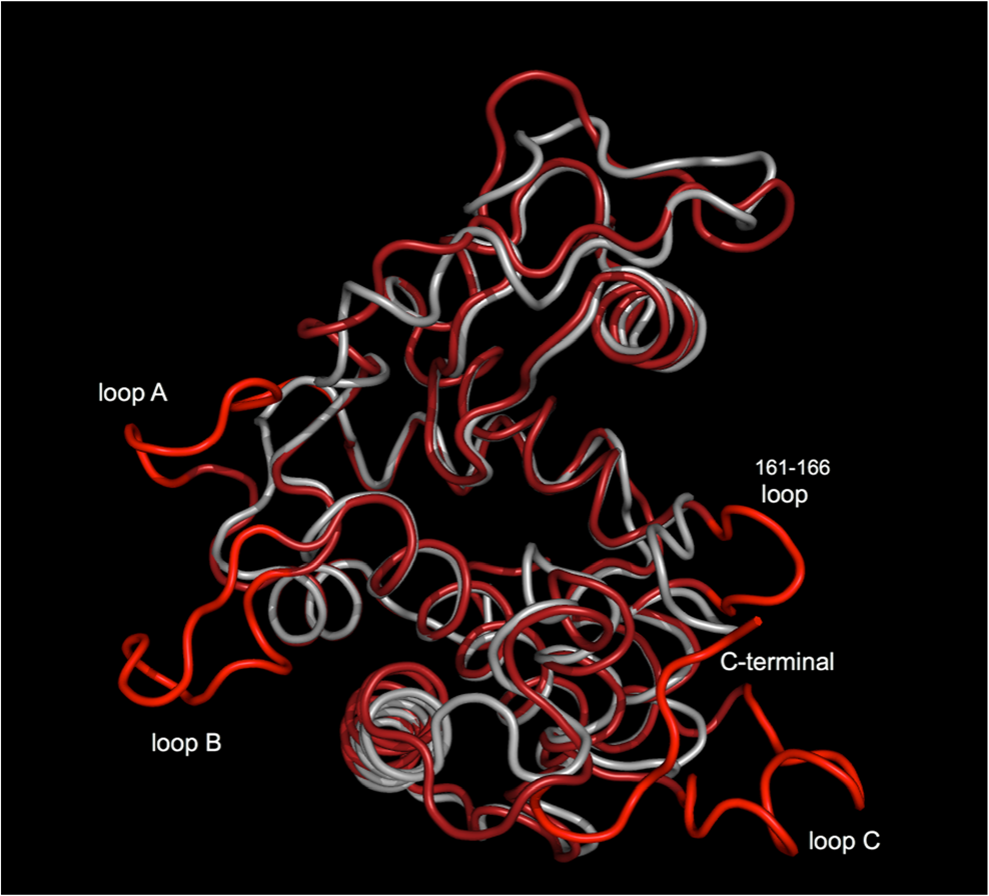
Structural superposition of GH19 ChiG from *Streptomyces coelicolor* (PDB file 2CJL) (light gray) and barley chitinase (PDB file 2BAA) (red)

This primal transfer event from plants to actinobacteria has been followed by selection events reflected by subtle structural differences among actinobacterial (and other bacterial) and plant GH19 chitinases. While the general structural skeleton is conserved among both GH19 groups (Hoell et al. 2006; Kezuka et al. 2006), the bacterial chitinases lack a *C*-terminal extension and several loops compared to plant enzymes (Fukamizo et al. 2009; Hoell et al. 2006; Ubhayasekera 2011) (Fig. 1). These structural variations between bacterial and plant GH19 chitinases may explain the preference of each enzyme on acting toward different forms of chitin substrates.

Few studies investigated the evolutionary relationships among enzymes with chitosanase activity. Extensive analysis of GH46 family suggested subdivision into five groups, A to E (Takasuka et al. 2004; Viens et al. 2015b). GH46 proteins are almost exclusively found in bacteria and chloroviruses. The recent publication of the whole genome sequence of the fungus *Lichtheimia ramosa* predicted the presence of two hypothetical GH46 genes (LRAMOSA04613 and LRAMOSA01487) which are the first and only GH46 members from Eukaryotes known so far (Linde et al. 2014).

Most actinobacterial GH46 chitosanases belong to group A (Viens et al. 2015b). However, a small number of genes from actinobacteria are found in group B, which is almost exclusively represented by chitosanases from *Bacillus* and related genera. The nucleotide composition analysis of the genes encoding the actinobacterial members of group B provided no clue of recent acquisition by HGT from bacilli to actinobacteria (Viens et al. 2015b; Zitouni et al. 2017). Alternative explanations such as recombination between actinobacterial GH46 genes or their ancestors (see below) could be suggested.

The GH19 chitinases and GH46 chitosanases both belong to a higher level of molecular hierarchy: the “lysozyme superfamily” which also includes families GH22, GH23, and GH24 grouping enzymes with lysozyme activity (Holm and Sander 1994; Monzingo et al. 1996). These enzymes share their 3D-fold and have common features in their catalytic mechanism. They act however on different substrates and display low amino acid sequence similarity (Hoell et al. 2010; Lacombe-Harvey et al. 2009; Monzingo et al. 1996; Wohlkönig et al. 2010). During their evolution, all these families could diverge from a common ancestor (Shakhnovich et al. 2005; Galperin and Koonin 2012). To derive the evolutionary relationships among members of the lysozyme superfamily, Wohlkönig et al. (2010) built a clustering tree by comparing 32 structures. They concluded that, from a structural point of view, these five GH families exhibit a continuum and are almost equidistant from each other. The ancestral fold has been conserved throughout evolution while the amino acid sequences have diverged, which led to functional diversification within the superfamily.

According to the data obtained from biochemical and site-directed-mutagenesis studies of the active site of a GH46 chitosanase (Boucher et al. 1995; Fukamizo 2000; Lacombe-Harvey et al. 2009; Marcotte et al. 1996; Robertus et al. 1998; Wohlkönig et al. 2010), it is conceivable that GH19 chitinases and GH46 chitosanases could arise from a less specialized common ancestor “half-chitinase, half-chitosanase.” Following this hypothesis, evolution of the chitinase function would occur in plants, resulting in formation of GH19 family which then was transferred to actinobacteria by HGT (Udaya Prakash et al. 2010). Evolution of chitosanase function would occur in Gram-positive bacteria (perhaps in parallel in high- and low-G+C branches) resulting in formation of the GH46 family. As only a few HGT from soil bacteria to plants are presently documented (Emiliani et al. 2009; Yang et al. 2015), this course of evolutionary events could explain why no GH46 chitosanase was identified so far in plants.

The chitosanases from family GH75 were studied at a much lesser extent. Despite numerous putative GH75 sequences identified in actinobacterial genomes, only one GH75 chitosanase from *Str. avermitilis* (Csn75A) has been the subject of characterization (Heggset et al. 2012). This family is abundantly represented in fungal and actinobacterial genomes. So far, only a few representatives were identified in other bacterial phyla. According to site-directed mutagenesis experiments performed on the fungal chitosanases from *Fusarium solani* f. sp. *phaseoli* SUF386 and from *Aspergillus fumigatus* (Chen et al. 2006; Shimosaka et al. 2005), GH75 chitosanases use aspartate and glutamate as catalytic residues. These two carboxylic catalytic amino acids are conserved throughout the members of family GH75 (data not shown). Because no GH75 chitosanase has been crystallized yet, one can only speculate about the structural properties of these enzymes. Thorough additional investigations regarding biochemical characterization and structure determination are still required before a model retracing the evolutionary history of GH75 could be proposed.

Recent advances in terms of enzyme evolution and superfamily functional diversity, and new analysis tools such sequence similarity networks (SSN) (Baier et al. 2016) will hopefully give rise to a better comprehension about the evolutionary history of enzymes which act on chitin and chitosan.

## Electronic supplementary material

ESM 1 (PDF 102 kb)

## Footnotes

1 In the review, “chitinolytic” will be used when no distinction is made between chitin and chitosan. When such distinction is necessary, the polymer “chitin” or “chitosan” will be specifically indicated.
