# Supplementary material for: Chitinolytic functions in actinobacteria: ecology, enzymes, and evolution
Source: Appl Microbiol Biotechnol. 2018 Jun 21;102(17):7219–30. doi: 10.1007/s00253-018-9149-4 (PMC6097792; doi:10.1007/s00253-018-9149-4)

Supplementary material

Applied Microbiology and Biotechnology

Chitinolytic functions in actinobacteria: ecology, enzymes and evolution

Marie-Ève Lacombe-Harvey, Ryszard Brzezinski and Carole Beaulieu

Département de biologie, Université de Sherbrooke, Sherbrooke (QC), J1K 2R1, Canada

Corresponding author: Carole Beaulieu, E-mail: Carole.Beaulieu@USherbrooke.ca,

phone: +1-819-821-8000 x.62997, Fax: +1-819-821-8049.

Supplementary Fig. S1

Streptosporangium roseum putative CsnR regulon

Methodology: The genome of *Streptosporangium roseum* DSM 43021 (GenBank accession number NC_013595.1) has been searched with RSAT tool using the consensus sequence AGGAAANTTTCCT and allowing two mismatches except at positions 5 and 9.
Only palindromes localized in positions adequate to their function as regulatory sequences were retained.
The following listing includes:
The name of the gene according to the GenBank file
The intergenic sequence preceding the ORF of a given gene
The putative protein family or function
CsnR boxes are marked in yellow
The translation initiation triplets are in blue and underlined

>Streptosporangium roseum GH8 putative chitosanase Sros_1551
GTACCGGCCCGCGATAATTTAGGCCCCCCATCCCCCTTGACCTGACATATCAGAAATGGT
GCCATCTTTCCCTAGTTAGGAATCTTTCCTAACTAGTTGATCGCAGAGAGGGCAGACCCT
GTG

>Streptosporangium roseum ROK protein, putative CsnR Sros_5819
CATCACCTTAGCAGGTCCGTCGAGATGTTCACCCCTCCCCTCGCCATCACAGGTTGATTG
CGGCACGCCCTTCACGCTTGACGGAATTCGCACGGGTTCCTTAGATTCACTCCGAGTTAG
GAAACTTTCCTAACTCCGTTTTACGGGCGACTGTATGACGTGTGCGGACACGGAGGACCT
GTG

>Streptosporangium roseum GH46 Sros_2053
GTGGAGCCGTGCCCCGGCTGCCGGCGCGCCGCCCGGGGCTCGGGCCGCCTTCGGGGGAAC
GCCCCCGATCGAGGGGAACGGGTTGACGGGCGGGGCATATCAGCGGTTAGGTTCGTTAGG
AAAGTTTCCTAACGAGCGGGCGAGGTTTTCTCGCCTCCGGTCCCCCCTCTGGAGAGACAC
GTG

>Streptosporangium roseum Chitin-binding protein Sros_0283
CGGCTCGCGGGTCCCTTCGACGGATCGGCGGGGCGCCGGCTCATGCCGGCGGCGAGCGGG
CCGACCGTGCCGGGGTCGCGTCCCCCCTGACAGGCCGGATGCGGCGGGGTTGGATGGATA
TTTACTGTTAGTAAAGTTTCCTTTATATTTGGTCCAACCCCCCAGCAAAGGAAGCATGAC
ATG

>Streptosporangium roseum Major facilitator protein Sros_0534
CATCGAGGCCGCCCGGGACCGCAGCCGCCAGCTCGCCGGGGGCTGACCGTCCCCCTCTAT
TAGGAAACTTTCCTGCATAAGGCATCATTTCCACAGAACTTCGGTGGGAATGAGGCAATC
GTG

>Streptosporangium roseum beta-hexosaminidase GH3 Sros_2183
GTGCGACATCCTGTCCGTCTCCCTCGTGGGTGACTTCTCTTCGGGCAGGCGACGGGGGCA
TGGGTCACCCCGGACAGACTTTTAATAGGAAAGTTTCCTGTCTATTTACGCAACCCGAAT
GCTACACATGTGGTACGCCTGAAACAAGCGACAAGTGGTGCACCGAATTTGGAGGCTTTG
ATG

>Streptosporangium roseum ROK protein Sros_2195
CGGGTGCCGGCGTGGTCCCCATGCCGCGCCACACCTGATCGAAGGCGCCTCCTCACGGTC
GGCGCTCTGACATCCCGCAGCCTGCTGTCACTCTTGCCTGCGGGCTGACTACGGTTAAAC
TAACAAGAAACTTTCTTAAAAGAAACGATCGTTAGCGGATCGTGACGGGGGGAGCCGATC
<u>TTG</u>

>Streptosporangium roseum chitinase Sros_2284
GAGCCGTACGGCGGCCAGGGCGGCTGCGAGATCGTGCGGCGGAAGGGATCGCACGCTCCG
TTGACCGCTATGCGGCAAATCCACGGAAGACCGTTGACGCTCAGTTCGCCCAGGTCTACG
ATCCGGGAAAACTTAAAGGAAAGTTTCCTATAAGTTATCCCCCCAGCAGTGGAGAAGCCC
<u>GTG</u>

>Streptosporangium roseum GH9 hydrolase SROS_2851
GGGTGACGGTCATGTTCCGCATGCTATTGACCGCCGCTCTTGACCCCCCTCCCAGGTGTC
GCTACCTTCCCGACAAAGGTTAGGAAGGTTTCCTAACTTCCCCCCGCACCAGGAGCGCCG
<u>ATG</u>

>Streptosporangium roseum Chitin binding protein SROS_3599
CCCAGTCCTACCCGACGGAGCCGACCGGTGGCCCCGCCGGGCGGGCCGGGTCTCGCCGCC
GAGGAGTCCGCGCAGGGCGCCGGGCATCCGGCGAAAGCCGGGGAACCGCATGACTAAAGC
ATTTACTGTTAGGAAACTTTCCTTTACGTTTGGCTTATCCCCCCAGTAAAGGAGTACCAC
<u>GTG</u>

>Streptosporangium roseum GH18 chitinase Sros_7134
TGGTCTCCGGCGTGCTGGGCATCGCCTGGCGCTGGCCGTAAACCTCTCTTCCCGCGATTG
GGCCCCCCGAAATTTCGCACCATTGACGGCGCGTGCGCGCGTGATTACGTTCACCTCAAA
TTGACAGTAAAGTTTCCTGTTAATTAACCCCCCAGTTCCAGCGCGTAGAGGCGGCGGCCC
<u>ATG</u>

>Streptosporangium roseum GH18 chitinase-like Sros_7591
CGTACGGCGCCGGCCGTCCGCCCGGCGTGTCCGGCGTGGTCGGCGGGGCCCTCGTGGCCG
TCGGATCCACCGCGCCCACCTCCGCTCGAACTGCGCCGTTTTGGGTCTTGTCGACATGGA
GCGCCAGGTTTACGATCCGCGTAATTGTTAGGAAACTTTCCTTTAAGAAAGAGTCCACGC
<u>ATG</u>

>Streptosporangium roseum GlcNAc transport system Sros_7707 Sros7706 Sros_7705
GTGCCGATCTGTCAGGGGAATGTCCTCCTTTGACGTTTGCTTTCTACCGGAAATGTCGCG
AGGACATTACGGCTCGGTCTAGACCGGCTGCGACCTCTTGACGGTGGACTCCAGGGGTAC
GTACCTTCGGGCAAACGTTTAGGAAACTTTCCTAATTGATCCCCCGTCCAGGAGGGTTAC
<u>ATG</u>

>Streptosporangium roseum chitin binding protein Sros_7908
GTCGCCTGCCGTACGGCCTCGCCGCCGCGGTCCCGCCGGCGACGGTAACGGCCGGCGGCG
GGGGGAGCGGGCTCCGGGACGCCGTGGGGTCATGACCGCGGACTCGGGAATCCTTCTTCG
GATGCGCCGGAACCTATGGCATCGGAGGCCGAATTTTTAGTAAGGTTTCTTGTTAATAAC
GTGCTATCCGTCGGCCGTTGGACATGTCCCACCTCCATCCCCCCTGTAGGAGGAGTCCAC
<u>GTG</u>

>Streptosporangium roseum GH18 chitinase Sros_8171
GCCCGTTTCATGTCCGGGGGCGCCCGGGGGGGCGTGGAACGAACCTTAAATCAGCCTTAT
ATCGATCGGTGAGGCATTGACCGCCCGGGAACGAGAATCTACGCTCTGGAAACTAATAGG
AAACTTTCCTATGAATTCGGGGGCTTGGCACTCTCTGACCTGGAGCAACCCTTGCGACGA
<u>ATG</u>

```
GH18_7591   CGATCCGCGT AATTGTTAGGAAACTTTCCTTTAAG AAAGAGTCCA
GH18_8171   ACGCTCTGGA AACTAATAGGAAACTTTCCTATGAA TTCGGGGGCT
CBP_0283    GATGGATATT TACTGTTAGTAAAGTTTCCTTTATA TTTGGTCCAA
ABC_7707    CCTTCGGGCA AACGTTTAGGAAACTTTCCTAATTG ATCCCCCGTC
GH18_2284   GATCCGGGAA AACTTAAAGGAAAGTTTCCTATAAG TTATCCCCCC
GH3_2183    CCGGACAGAC TTTTAATAGGAAAGTTTCCTGTCTA TTTACGCAAC
CBP_3599    CTAAAGCATT TACTGTTAGGAAACTTTCCTTTACG TTTGGCTTAT
GH18_7134   GTTCACCTCA AATTGACAGTAAAGTTTCCTGTTAA TTAACCCCCC
MFP_0534    GACCGTCCCC CTCTATTAGGAAACTTTCCTGCATA AGGCATCATT
CBP_7908    TCGGAGGCCG AATTTTTAGTAAGGTTTCTTGTTAA TAACGTGCTA
Ron_2195    ACTACGGTTA AACTAACAAGAAACTTTCTTAAAAG AAACGATCGT
GH8_1551    GCCATCTTTC CCTAGTTAGGAATCTTTCCTAACTA GTTGATCGCA
GH9_2851    CCTTCCCGAC AAAGGTTAGGAAGGTTTCCTAACTT CCCCCCGCAC
GH46_2053   CAGCGGTTAG GTTCGTTAGGAAAGTTTCCTAACGA GCGGGCGAGG
ROK_5819    TAGATTCACT CCGAGTTAGGAAACTTTCCTAACTC CGTTTTACGG
```

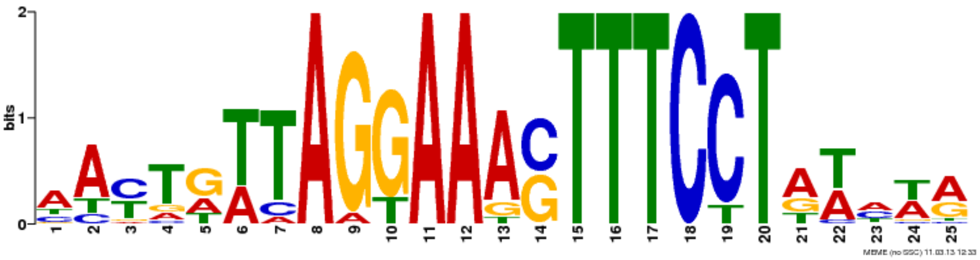

Supplement: Supplementary file 1 — (PDF 102 kb) [file 253_2018_9149_MOESM1_ESM.pdf]
